# Survival guide: Escherichia coli in the stationary phase

**Published:** 2015

**Authors:** P. Pletnev, I. Osterman, P. Sergiev, A. Bogdanov, O. Dontsova

**Affiliations:** Moscow State University, Chemistry Department, Moscow, 119991, Russia

**Keywords:** stationary phase, stress, starvation, survival, Escherichia coli

## Abstract

This review centers on the stationary phase of bacterial culture. The basic processes specific to the stationary phase, as well as the regulatory mechanisms that allow the bacteria to survive in conditions of stress, are described.

## INTRODUCTION

The conditions that sustain constant bacterial growth are seldom found in nature, in contrast to when bacteria are cultured under optimal laboratory conditions in rich media and at an optimum temperature. The influence of harsh environmental factors, accumulation of toxic metabolic waste products during starvation, and antibiotics – all this threatens the survival of *Escherichia coli *and other bacteria. For protection against harsh environmental influences, bacterial culture can enter a stationary phase where its internal systems of protection against stress become activated. In order to survive under adverse conditions, the bacterial culture can dramatically change its organization both at the molecular and cellular levels.

Knowledge of the processes occurring in the stationary phase is necessary for both fundamental and practical viewpoints. Cells in the stationary phase are orders of magnitude more resistant to antimicrobials and acquire the ability to survive even under extremely adverse environmental settings. This review focuses on the basic processes characteristic of the stationary phase.

## PHASES OF BACTERIAL CULTURE GROWTH

The growth of a bacterial culture represents a process of sequential division of the cells of the culture to form two identical daughter cells.

**Fig. 1:**
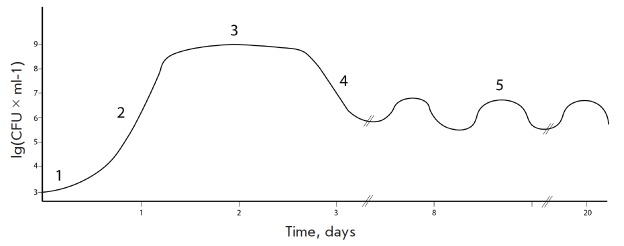
Bacterial growth curve. 1 – lag-phase, 2 – logarithmic phase, 3 – stationary phase, 4 – death phase, 5 – long-term stationary phase

Study of *E. coli *cells survival during cultivation for several days revealed a characteristic growth curve pattern comprising five phases. Despite differences in growth conditions, measurements, and even speciesspecific features, the general shape of the curve always remains the same, except for some parameters (*Fig. 1*) [1].

The moment when the cells enter into the nutrient medium after being in the stationary phase, one can observe what is usually called the lag phase. This phase is characterized by an almost absence of bacterial growth in culture for some time, which can be attributed to the need for adaptation of cellular metabolism to the new habitat conditions. The duration of the lag phase is determined not only by the bacterial species, but also by the length of time that the cells have remained in starved conditions [2].

Once the cells are adapted to the new cultivation conditions, they start to divide exponentially and enter a logarithmic growth phase. Since bacterial cells divide asexually by binary fission, an increase in the number of cells in the medium per unit of time is well approximated by an exponential function. The growth rate of culture in the logarithmic phase is characterized by the number of doublings. It is worth noting that this rate depends directly on the culture medium, being slower in nutrientpoor conditions and faster in nutrient-rich conditions. The standard laboratory strain *E. coli *MG1655 K-12 has a doubling time of about 30 min at 37°C.

Once nutrients in the medium are exhausted, bacterial culture enters a stationary phase, which is characterized by equilibrium between the numbers of dividing and dying cells and represents a plateau in the growth curve. It should be noted that the term “stationary phase” refers specifically to the region on the growth curve which is characterized by equilibrium between dividing and dying cells, but not to the mechanism of defense during starvation. The stationary phase begins in the cell population not only due to the exhaustion of the external environment, but also because of the various other stress factors. Over time, cultures in the stationary phase accumulate toxic products of catabolism in the environment, leading to a decline in the number of viable cells, known as the death phase. Stochastic death and programmed cells death have been postulated to be responsible for this phase.

The end of the death phase comes after the majority of the population dies and the dead cells release nutrients into the environment. Survivors may use these substances for their survival that brings the bacterial culture to a state of long-term stationary phase, with viability remaining for several weeks and even months. One of the characteristics of the long-term stationary phase is the successive increase and decrease in the titer of viable cells in the population. This phenomenon is referred to as the growth advantage in the stationary phase (GASP) phenotype and is explained by the fact that mutant cells, which are more adapted to grow under these conditions than the parent strain, appear among the bacterial population [3].

## CHANGES IN DNA STRUCTURE AND TOPOLOGY

Genomic DNA of *E. coli *is represented by a single circular chromosome, which forms a structure in the cytoplasm of bacteria called the nucleoid. This structure also includes nucleoid proteins (regulatory and structural) and RNA [4].

A multitude of proteins are responsible for maintaining the nucleoid structure, whose expression depends on the growth phase of the bacterial culture. The IHF, HU, Dps, Fis, and H-NS proteins are considered the most essential structural proteins of the nucleoid.

The active form of H-NS is a dimer characterized by the presence of two oppositely directed DNA-binding domains making it possible for the protein to act as a “bridge” between two DNA duplexes. H-NS is not sequence- specific, but it exhibits greater selectivity for bent DNA rather than linear DNA [4]. In the logarithmic growth phase, one molecule of H-NS in the cell accounts for 1,400 bp DNA [5].

The integration host factor (IHF) is a heterodimeric protein exhibiting specificity for consensus regions of DNA of approximately 30 bp. Binding of IHF causes DNA bending, which is stabilized by the interaction of the negatively charged DNA backbone and the mostly positively charged surface of the protein. It is shown that IHF binding can reduce the length of the DNA by 30% [4]. Expression of IHF is at a maximum in the stationary phase, and one molecule of IHF accounts for 335 bp genomic DNA [5]. Probably, IHF is responsible for the organization of the nucleoid structure in the early stationary phase.

The histone-like HU homodimer protein is composed of either two HUα or two HUβ subunits. HU exhibits a high (40%) structural similarity to the protein IHF. HU binds to DNA nonspecifically, but it has selectivity for overwound and unordered DNA. HU seems to be able to induce and stabilize the bend of the double DNA helix with a variety of angles of rotation. Random HU binding leads to a large number of “mobile” bends of DNA (with bending angles of 180°), which eventually reduces the length of linear DNA by 50% [4]. HU content is highest in cells during the logarithmic phase, where one molecule of the protein accounts for approximately 550 bp DNA [5].

The factor for inversion stimulation (Fis) is a homodimer DNA-binding protein capable of recognizing certain consensus sequences of 15 bp in length, but it can also efficiently bind DNA at random sites. Binding of Fis produces DNA bending by 50–90°. Many Fis binding sites are located in the promoter regions of operons, where binding of the protein plays a regulatory role. Fis is believed to be the “sensor” of DNA supercoiling. Depending on the topology of DNA, Fis exhibits the ability to down regulate the expression of the DNA gyrase gene [4]. Fis is one of the structural proteins of the nucleoid most represented in the logarithmic phase, the content of which reaches 1 molecule per 450 bp DNA [5].

In the stationary phase, the nucleoid becomes more condensed to protect DNA from damage. This mechanism is implemented by means of the Dps protein (DNA-binding protein from starved cells), which is capable of non-specific binding with DNA and is active exactly in periods of starvation [6]. Under oxidative stress in the logarithmic phase, the expression of the Dps-encoding gene is under the control of σ^70^-subunit of RNA polymerase and OxyR protein, and during periods of starvation it is regulated by σ^38^-subunit [7]. After the induction of synthesis in the stationary phase, Dps becomes the most represented protein in *E. coli *cells [7]. Monomers of Dps form ring-like dodecamer structures, which bind to DNA in the presence of Mg^2+^ and promote the formation of a highly ordered and stable nucleoprotein complex called “biocrystal” [8]. It is the formation of this complex that leads to the condensation of the nucleoid. It seems that the global protective role of Dps against different forms of stress (starvation, oxidative stress, UV and γ-irradiation, thermal stress and pH) is implemented via a combination of several of its properties: the ability to condense DNA, chelate iron ions, and exert ferroxidase activity, as well as the ability to regulate gene expression [6, 9].

CbpA (Curved DNA binding protein) is another protein which helps protect DNA from damage in the stationary phase. In the log phase, CbpA is absent in the cells, but upon onset of the stationary phase its amount increases up to 10,000 copies per cell. Transcription of the *cbpA *gene also depends on σ38-subunit of RNA polymerase [5].

CbpA binds DNA being in the form of a dimer, resulting in DNA compactization. This complex protects DNA from *in vitro *degradation by endodesoxyribonucleases [10].

**Fig. 2:**
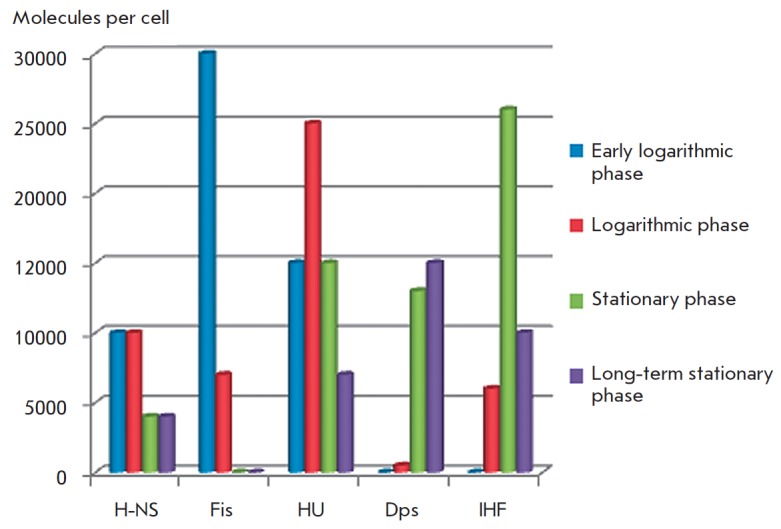
Normalized (H-NS, Fis, HU, IHF – dimers, Dps – dodecamers) amounts of structural nucleoid proteins during different growth phases. Based on data from [5]

The structural proteins of the nucleoid influence directly not only the structure of the bacterial chromosome, but they are also actively involved in the regulation of gene expression. It should be noted that these proteins have similar functions in compactization and protection of DNA from damage, and the prevalence of each of them depends on the growth phase (*Fig. 2*). However, due to differences in the mechanisms and ways of compactization the use of either structural protein of the nucleoid allows the cell to adapt, making access to DNA easy under the most favorable conditions and providing maximum protection of the genetic material against stress.

It has been shown recently that the methylation of cytosine residues in bacterial DNA can influence the regulation of protein synthesis in the stationary phase. In the study of a DNA methyltransferase Dcm gene knockout strain, it was found that the strain exhibits considerably increased synthesis of proteins of the stationary phase, and particularly, the RNA polymerase σ^38^-subunit [11].

## TRANSCRIPTIONAL REGULATION IN THE STATIONARY PHASE

***σ*^38^ – Stationary phase sigma-factor**

Transcription is a central and vital process when RNA is synthesized on a DNA template. This reaction is catalyzed by RNA polymerase.

In bacteria, transcription is initiated by the RNA polymerase holoenzyme that is formed by a multisubunit (α_2_ββ’ω) core, which contains the active center and performs RNA synthesis, and the initiation factor – σ-subunit, or σ-factor. In order to form an active holoenzyme capable of synthesizing RNA, σ-subunit, which is responsible for promoter recognition, must bind to RNA polymerase [12]. It is known that the *E. coli *genome encodes seven different σ-factors (*Table*), each capable of recognizing only a certain group of promoters. It is the σ-subunit which is the primary regulator of cellular transcription. The use of different σ-factors allows the cell to dramatically change their transcriptome in response to various signals.

**Table:** List of σ-factors of E. coli

Sigmafactor	Function	Reference
RpoD (σ^70^)	Housekeeping gene expression	[13]
RpoS (σ^38^)	Initiation of the stationary phase and stress response	[14]
RpoF (σ^28^)	Synthesis of flagella andchemotaxis	[15]
RpoN (σ^54^)	Activation of nitrogen metabolism	[16]
RpoH (σ^32^)	Response to heat shock	[17]
RpoE (σ^24^)	Response to stress associated with membrane damage	[18]
FecI (σ^19^)	Expression of nitrate transport genes	[19]

Under the conditions of the stationary phase, a bacterial cell has to regulate transcription in such a way as to activate the expression of the genes required for survival under stress and starvation and to suppress the transcription of “unnecessary” genes. *E. coli *uses σ^38^(σ^S^)-factor encoded by the *rpoS *gene for this purpose, which acts as the main regulator of transcription in response to various forms of stress. Genome-wide analysis of gene expression dependent on σ^38^-factor showed that σ^38^ directly or indirectly regulates the transcription of about 10% of *E. coli *genes [20].

σ^S^-factor is responsible for the transcription of the genes involved in stress response and secondary metabolism. Most of the genes regulated by σ^38^-subunit undergo additional regulation. This factor is required for the transition of the bacterial culture to the stationary phase. There are some σ^38^-dependent genes whose expression is maximal at the time of transition of the cultures from the exponential into the stationary phase [14].

σ^S^-factor is a homologue of the main cellular sigma factor –σ^70^, which is responsible for the transcription of housekeeping genes and has the highest affinity for RNA polymerase. It is shown that σ^38^-subunit recognizes the same consensus sequences as σ^70^, the most significant of which are the -10 and -35 elements. It is assumed that the difference between the promoters that are recognized by these sigma factors consists in singlebase substitutions in the region of consensus hexamers: i.e., σ^38^ specificity can be determined by a small deviation of the hexamer sequence from the consensus sequence [21]. σ^38^-RNA polymerase is reported to be selective for promoters with non-optimal sequences for σ^70^ at the region of the -10 and -35 elements [22]. Based on the known promoter sequences, it was shown that an A/T-rich region in the -10/+1 region can improve promoter recognition by σ^38^-RNA polymerase [14].

**Fig. 3:**
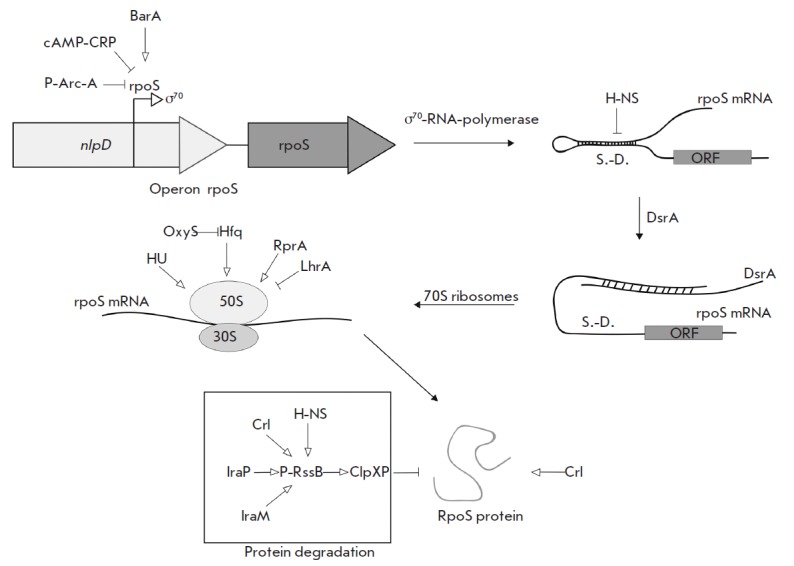
Regulation of σ^38^ expression and activity. The upper left side of the figure shows the regulation of σ38 (*rpoS*) gene transcription. Effect of transcription regulatory proteins shown by arrows. The right-hand part of the figure illustrates the effect of the H-NS protein and DsrA RNA on the secondary structure of *rpoS *mRNA. Shine-Dalgarno sequence designated as “S.-D.”, open read frame designated as ORF. Proteins affecting the translation of *rpoS *mRNA are shown in the center of the picture. Proteins affecting the stability of σ38-protein are shown in the lower part of the picture

Expression of σ^38^-subunit undergoes complex regulation at all levels (transcription, translation, factor activity, stability of σ^38^ and its mRNA), which apparently allows for enhancement of the sensitivity of the cellular response to a variety of stress signals (*Fig. 3*).

*rpoS *transcription is repressed by the phosphorylated form of the ArcA regulator [23], and a complex of cAMP-CRP also represses transcription [24]. The BarA protein is necessary for inducing expression in the logarithmic phase [25]. The signaling molecule pp- Gpp positively influences the basal level of synthesis of σ^S^ [26]. The expression of *rpoS *increases by an order of magnitude in the transition from the logarithmic to the stationary phase [24].

It was shown that a multitude of factors influence the secondary structure, stability, and ability of the* rpoS *mRNA of translation. The *rpoS *gene mRNA contains a long 5‘-UTR [27], which plays an important role in the regulation of translation and stability of this mRNA. The regulator H-NS binds to mRNAs and promotes its decay [28]. In turn, the small DsrA RNA stabilizes mRNA and promotes translation initiation via unfolding of the secondary structure of the mRNA in the ribosome entry site region [29]. The Hfq protein is necessary for translation of *rpoS *mRNA [30], and small OxyS RNA represses translation of σ^S^-subunit most likely due to changes in the activity of Hfq [31]. Small RprA RNA and HU protein stimulate translation of σ^38^-subunit. Under phosphorus starvation, σ^S^ accumulates due to increased amounts of *rpoS *mRNA. Protein LrhA, together with Hfq, is able to repress the translation of *rpoS *mRNA [32].

It is known that 6S RNA activates transcription from certain σ^S^-dependent promoters without influencing the level of *rpoS *expression [33]. The signaling molecule ppGpp enhances the ability of σ^S^ to displace σ^70^ from the minimum enzyme RNA polymerase [34]. It has been shown that the protein Rsd performs a similar function [35]. Lack of nitrogen leads to the expression of genes regulated by σ^S^; however, the level of expression of the subunit itself increases only 2-fold: i.e., nitrogen scarcity apparently influences the activity of the sigma factor more than its expression [36]. RNA polymerase assembly factor Crl increases the activity of σ^S^ , influencing its ability to bind RNA polymerase [37].

In the logarithmic growth phase, σ^S^-subunit is degraded by energy-dependent ClpXP protease, which instantly cleaves factor σS under excess energy in the cell [38]. Degradation of σ^S^ by ClpXP protease requires an additional protein, RssB, to promote rapid proteolysis of σ^S^. RssB recognizes σ^S^-factor. Poly (A)-polymerase and the IraP, IraM, H-NS, and Crl proteins enhance the RssB-mediated effect [37, 39-41]. Transcription of* rssB *is under the control of σ^S^. Lack of carbon sources leads to the accumulation of the sigma factor due to an increase in its stability. The molecular mechanism of this process has not been investigated [42].

In summary, it should be noted that *rpoS *is expressed in response to sudden adverse changes in environmental conditions. In this, the complex system regulating the expression of this gene and momentary decay of σ^S^ under optimal growth conditions permit the cell to efficiently change its transcriptional profile in response to stress and quickly return to the use of σ^70^ when adverse conditions no longer exist.

**Some transcription regulators in the stationary phase**

Transcriptional regulation in the stationary phase is not limited to the change of sigma factors. The bacterial cell contains many regulators that are specific to the stationary phase to alter the expression of certain genes.

One of these regulators is a highly conserved bacterial protein, Lrp (leucine-responsive regulatory protein), which can act both as a transcriptional repressor and activator. This protein is one of the main regulators in the stationary phase influencing more than 400 genes of *E. coli*, with about 75% of these genes active exactly in the stationary growth phase. Among these are genes whose products are responsible for the biosynthesis of amino acids, catabolism, the transport system of nutrients, pili synthesis, and the use of various carbon sources [43]. The main role of Lrp is adaptation of cellular metabolism to environmental settings. Interestingly, Lrp increases the amino acid anabolism level but reduces the level of their catabolism [44].

*lrp *gene expression is upregulated by means of a signaling molecule, ppGpp. Binding of leucine may also affect its activity. Lrp is able to activate the expression of the genes required during starvation, and to repress genes that are active in the logarithmic growth phase. It is assumed that the mechanism of sensitivity to starvation is based on the binding of leucine molecules, reduction in the intracellular concentration of which may be a sign of this condition [45].

Mutations that disrupt the function of the DNA binding domain of Lrp enhance the effect of GASPphenotype, in particular, because the cells with this mutation are able to more efficiently metabolize certain amino acids [3].

Not only protein regulators, but also small RNAs affect gene expression in the stationary phase. These RNAs can stimulate translation and affect the stability of specific mRNAs. The genome of *E. coli *contains more than 60 genes of small RNAs, a part of which is responsible for the regulation of the stress response. Bacterial small RNAs are short RNAs of 80–100 nucleotides. The activities of many of them require binding to the chaperone Hfq [46] capable of forming a complex with AU-rich regions of RNAs, whereby it can stabilize the mRNA or, alternatively, enhance the hydrolysis and inhibit its translation. Small DsrA and RprA RNAs stimulate translation of σ^S^-factor. Under optimal growth conditions, the 5’-UTR of *rpoS *mRNA has a secondary structure that blocks the ribosome entry site. Small RNAs of DsrA and RprA are capable of interacting with the 5’-UTR of *rpoS *mRNA via complementary regions, which changes the secondary structure of mRNA and opens the ribosome entry site [47].

Another small RNA, OxyS, appears under oxidative stress and represses translation of RpoS by competitive binding of RNA chaperone Hfq [31]. The other small RNAs active in the stationary phase are MicA and RybB, which are involved in the regulation of the outer membrane permeability. It is the outer membrane that serves as the first line of defense in the contact with the environment. To protect the cells from damage, the composition of the membrane changes to allow the cell to endure periods of stress. MicA and RybB with Hfq are understood??? to cause antisense inhibition of translation. RybB RNA controls the expression of two proteins, the outer membrane components – OmpC and OmpW. In turn, the small MicA RNA causes the decay of mRNA of the OmpA outer membrane protein [48].

**The stringent response**

Inhibition of rRNA synthesis under amino acid starvation was one of the first mechanisms of gene expression regulation in bacteria ever described at the molecular level. Genetic analysis made it possible to identify a mutation that leads to the absence of reduced rRNA synthesis in response to amino acid starvation. This mutation was described as “attenuating the strictness” of the influence of the number of amino acids on the biosynthesis of RNA [49]. Later, it was shown that this mutation inactivates the *relA *gene, encoding (p)ppGpp- synthase [50]. It was shown further that the synthesis of this nucleotide regulator is a response of the cell to stress. This regulatory system controls replication, translation, transcription, and the activity of the enzymes of the stress response [51].

Synthesis of ppGpp is performed by two proteins with similar functions – RelA and SpoT. RelA, or pp- Gpp-synthase I, only synthesizes guanosyltetraphosphate, while SpoT shows double catalytic activity: pp- Gpp synthesis (ppGpp-synthase II) and its degradation (ppGpp-hydrolase). The activity of RelA and SpoT is regulated by different mechanisms. RelA is responsible for the transmission of the starvation signal of one or more amino acids, and SpoT senses carbon, phosphorus, iron, or fatty acids scarcity [3].

Under the abundance of nutrients, RelA is associated with 70S ribosomes. In the case of amino acid starvation, deacylated tRNA accumulates in the cell. This tRNA, being in excess, can enter into the A-site of the ribosome, the ribosome then stops, which leads to dissociation of the RelA ribosome complex. In the free form, RelA is capable of catalyzing the transfer of pyrophosphate from ATP or GTP to GDP [52]. One feature of this mechanism is that RelA responds to the absence of a single amino acid, even if other amino acids are present in sufficient amounts [50].

**Fig. 4:**
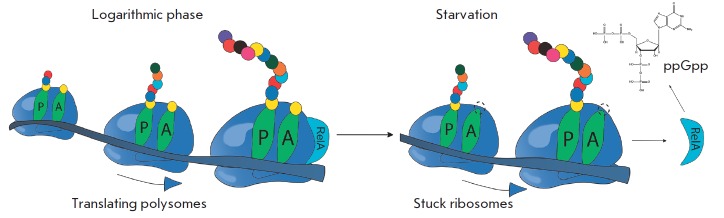
Scheme of RelA-dependent synthesis of ppGpp during amino acid starvation

It is known that a bacterial cell contains very few molecules of RelA that, for a long time, could not be correlated to the experimentally observed rate of pp- Gpp accumulation under amino acid starvation. It was proposed that when stuck ribosomes appear and RelA dissociates from ribosomes, a single act of dissociation is accompanied by the synthesis of the ppGpp molecule. After this, free RelA can “hop” to the neighboring ribosome, translating mRNA. If it is also unable to conduct the synthesis because of deacylated tRNA in the A site, the cycle is repeated, and if it is active, RelA remains bound to this ribosome in the inactive form (*Fig. 4*) [52].

Under favorable growth conditions, SpoT exerts only hydrolytic activity towards ppGpp, which leads to absence of guanosyltetraphosphate in the cell. The hydrolytic activity of SpoT is repressed upon binding to deacylated tRNAs. Because of this mechanism of activation, SpoT possesses sufficient activity only under deficiency of a large number of different amino acids. SpoT is believed to be associated with an acyl-carrying protein that allows it to control the amount of fatty acids in the cell. Probably, this mechanism also allows it to “feel” carbon starvation [53]. At a low concentration of fatty acids, synthesis of ppGpp is induced. With the help of the DksA partner protein, ppGpp binds to β-subunit of RNA polymerase, directly affecting the affinity to different promoters and thus altering the expression level of more than 80 genes. Particularly important is the suppression of the expression of all components of the protein biosynthesis system: rRNA, ribosomal proteins, and translation factors [54].

ppGpp, together with antisigma factor Rsd, helps σ^38^-subunit to compete for the enzyme base of RNA polymerase by reducing the affinity of σ^70^ to RNA polymerase. When environmental conditions become favorable, the hydrolytic activity of SpoT is restored and the ppGpp level decreases, which means the end of the stringent response [34].

**Translation process in *E. coli *in the stationary growth phase**

Protein synthesis is one of the most important processes in the cell. The key actor here is the ribosome; a compound nucleoprotein complex capable of synthesizing proteins according to information encoded in mRNA. In the stationary phase of a bacterial culture, there is a sharp decrease in the level of protein synthesis, which is not surprising, since translation is considered the most energy-consuming processes in the cell, and under deficiency of amino acids and other resources in a bacterial cell it is necessary to suppress translation. It should be noted that the processes affecting translation in the stationary phase are highly dependent on the duration and grade of the state of starvation. In response to starvation, a variety of mechanisms to rescue a single cell and, later, the entire bacterial population, are activated.

**Defense mechanisms at minor starvation**

When nutrients in the cell are exhausted, deacylated tRNA and truncated mRNA accumulate. Ribosomes can become stuck on these mRNA, since during the synthesis the ribosome reaches the end of mRNA and does not find a stop codon. This ribosome remains bound to mRNA and cannot be released due to the fact that the mechanism for termination of translation depends on the presence of a stop codon. This ribosome can be “rescued” through the mechanism of trans-translation.

Trans-translation is performed by a complex of an unusual tmRNA (transport and messenger RNA) with a small protein, SmpB. tmRNA consists of two domains: the tRNA-like domain (TLD) and mRNA-like domain (MLD). The similarity of the TLD-domain and tRNA is not only structural, but also functional. This domain is recognized by alanyl-tRNA synthetase, and the 3’-end of tmRNA is charged with an alanine residue [55].

In the case of stuck ribosomes, cooperation between tmRNA, SmpB, and the EF-Tu elongation factor allows the cell to recognize these ribosomes [56], after which the complex SmpB and tmRNA enters the A-site of the ribosome, where SmpB takes a shape mimicking the anticodon structure of alanine tRNA. Then, by means of hydrolysis of the GTP molecule the peptide from the tRNA in the P-site is carried to the alanine residue of tmRNA in the A-site, and translation resumes on the MLD-domain template of tmRNA [56]. C-terminal peptide is encoded in the MLD, signaling the need for degradation of this polypeptide chain. As a result of trans-translation, the stuck ribosome is freed and the potentially harmful polypeptide is degraded by proteases [57].

Another way to rescue a ribosome which has arrived at the 3’-end of mRNA and has not met a stop codon is the use of the ArfA and ArfB proteins. The *ArfA *gene encodes a short polypeptide consisting of 72 amino acid residues. The functional form of the protein consists of 55 amino acids and is translated from a fragment of the mRNA truncated by the ribonuclease III. Due to the lack of a stop codon, emergence of this polypeptide is possible only in case of a disturbed trans-translation mechanism. ArfA binds to the stuck ribosome and recruits the RF2 termination factor thereto, which leads to cleavage of the polypeptide chain from the peptidyltRNA and ribosome release [58]. Factor ArfB also acts similarly, binding to the empty A site of ribosomes and catalyzing the hydrolysis of peptidyl-tRNA independently of translation termination factors [59].

**Hibernating ribosomes as a response to growing starvation**

Synthesis of ribosomes is a highly energy- and resource- consuming process. That is why there must be mechanisms to suppress translation impermissible for starved cells, but all the while retain existing ribosome until better times.

It turns out that a mechanism for storing inactive ribosomes does exist and represents a temporary “switch off” of ribosomes. This process, referred to as ribosome hibernation, is activated within the stringent response, when deacylated tRNAs accumulate in the cell under a deficiency of amino acids, which serves as a signal to the synthesis of the molecule ppGpp using the enzyme RelA associated with ribosomes [52]. It is ppGpp that regulates the expression of the genes that encode the proteins of ribosome hibernation.

**Fig. 5:**
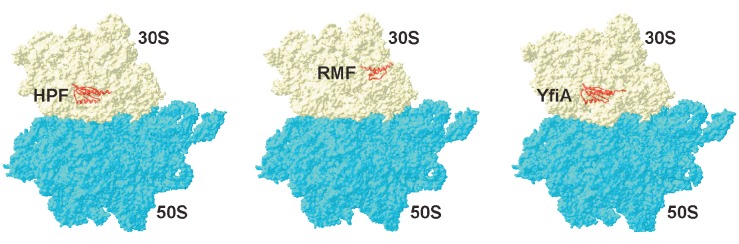
Binding sites of the hibernation factors RMF (PDB: 4V8G), HPF (PDB: 4V8H), and YfiA (PDB: 4V8I)

The main route of hibernation is the formation of “sleeping” 100S dimers and 70S inactive monomers from active 70S ribosomes. HPF, RMF, and YfiA are the major hibernation proteins of *E. coli *(*Fig. 5*).

The first two proteins are responsible for the formation of inactive 100S dimers. In the first step, the RMF protein binds to the 16S rRNA that interacts with the Shine-Dalgarno sequence of the mRNA. It is this area that is critical for the initiation of translation in prokaryotic cells, and the binding itself of this protein inhibits translation. It is particularly important that the RMF cannot bind to the translating ribosomes that prevent formation of under-synthesized proteins that may be toxic to a cell. Binding of the hibernation factor in this region leads to a rotation of the head of the small subunit. This change in conformation contributes to the formation of 90S ribosomal dimers. After dimerization of two ribosomes and 90S dimer formation, the HPF hibernation factor binds, which further stabilizes this structure and closes access for tRNA to the A- and P-sites of ribosomes. The resulting complex of two 70S ribosomes, together with the HPF and RMF proteins, has a sedimentation coefficient of 100S. Dimerization of ribosomes is reversible. When a cell enters a nutrientrich environment, the complex of hibernation factors and ribosomes dissociates to form two active 70S ribosomes [55, 60].

Another path of ribosome hibernation is binding of the Y (YfiA) protein by 70S ribosomes, which leads to the formation of inactive monomers 70S. The N-terminal part of the Y protein is similar to protein HPF; it also binds within the entry site of tRNA. Moreover, the C-terminus of the protein prevents RMF protein binding to the anti-Shine-Dalgarno sequence on 16S rRNA. After binding to the ribosome, YfiA inhibits its activity and prevents dissociation into individual subunits. When a cell enters favorable settings, the Y protein leaves the ribosome and translation restarts [60].

Literature data provide evidence of the complex influence of the stringent response on protein synthesis in a bacterial cell. Under the effect of ppGpp, not only is the expression of the biosynthetic apparatus suppressed, but translation itself is inhibited via hibernation factors.

**Translation under conditions of prolonged starvation. Death phase and programmed cell death**

When nutrients almost completely disappear from the environment and a critical amount of toxic metabolic waste products accumulate, programmed cell death (PCD) is activated in order to destroy most of the population and retain a small number of living cells. The population by this “sacrifice” reduces the load on the remaining bacteria, which will resume their generation when new resources appear.

This is the translation system which is particularly sensitive to intensifying starvation. As already mentioned, using the components of the translation machinery, in particular, ribosomes, a starvation signal is transmitted to all components of a bacterial cell [55]. The PCD mechanism is based on a toxin-antitoxin (TA) system: a set of two genes that together encode both a protein “poison” and a corresponding “antidote” antitoxin. Degradation of the antitoxin and downregulation of its expression leads to accumulation of active toxin and cell death.

The most studied and important unit of TA is the* mazEF *system revealed in a multitude of prokaryotes. This unit consists of two genes located in the same operon – *mazF *and *mazE*. The first gene encodes a stable cytotoxic protein and the second a labile antitoxin protein, which is easily degraded by ATP-dependent protease ClpAP. Under normal conditions, genes of both proteins are expressed, which does not allow the toxin to influence the cell because of the formation of a toxin-antitoxin complex. The starvation activates the synthesis of ppGpp, which suppresses transcription of the *mazEF *operon. After that, mazE is rapidly degraded and mazF is released. Toxicity of mazF is caused by its endoribonuclease activity, specific for the ACA sequence in mRNA, as well as a 3’-end fragment of the 16S rRNA [61]. After cleavage of the 3’-end of 16S rRNA, ribosomes “lose touch” with the anti-Shine-Dalgarno sequence that drives the translation of canonical mRNA. It has been previously shown that these ribosomes exhibit high selectivity towards the synthesis of small proteins, including both “death” proteins, killing the cell and the proteins necessary to retain small cell populations. It is assumed that in this way the system* mazEF *mediates programmed cell death, leading to the death of most of the population and the continued survival of a small sub-population [62].

Later, it was showed that this system of PCD is regulated by the signal peptide referred to as EDF (Extracellular Death Factor) and having the sequence NNWNN. This peptide is a component of a system of intercellular communication among bacteria called quorum sensing (QS). At a critical population density in a bacterial culture, EDF peptide, which is able to easily penetrate the cells, appears. This peptide significantly increases the activity of mazE and reduces the ability of mazF to inhibit the toxin. Thus, programmed cell death is activated in all cells in which EDF enters, and only a small population of cells remains intact and survives [63].

**Structural features of cells in the stationary growth phase**

The transition into the stationary growth phase, as mentioned above, is accompanied by the accumulation of factor σ^38^. Transition to σ^38^ influences not only metabolic and regulatory pathways, but also dramatically alters the physiology of the bacterial cell. It has been shown that the genes whose expression is controlled by σ^38^ are involved in the change of cell morphology, stress response, metabolic adaptation to starvation, and longterm survival in the stationary phase.

The bacteria in the stationary phase undergo adaptation of morphology critical for survival. The cells become smaller as a result of two processes: reductive division and formation of dwarf cells [64].

Reductive division is caused by the fact that the processes of DNA replication and cell division are initiated at the time of entrance of cells into the stationary phase, when, due to deficiency of resources, further growth of the cells is blocked at all levels of regulation. The cells cannot divide in the stationary phase, and because of the possibility of accidental initiation of DNA replication, cells with a duplicated number of chromosomes appear. As a result, the bacterial culture in the stationary phase becomes extremely heterogeneous in its chromosomal composition. Bacterial cells may comprise even more than two chromosomes [65]. The reason for the heterogeneity of the cell by the chromosome content may be that, in some cells during growth, a minimal distance between the nucleoids had not been achieve, which does not allow the cell division machinery to act. Another reason may be that, in the stationary growth phase, the termination of replication and DNA decatenation processes are disrupted in the cells [65]. When considering cell survival, mutation rate, and genomic stability, one should consider the fact that the cells in the stationary phase contain different numbers of chromosomes. The cells formed by reductive division are saphenous in morphology. This morphological feature could be explained by the influence of the* bolA *gene, which is actively expressed in the stationary phase under the control of RpoS.

It is most likely that the influence of BolA on cell morphology is determined by the transcription regulation of the *dacA *(gene encoding the penicillin binding protein 5, PBP5), *dacC *(a gene coding for a penicillin binding protein 6, PBP6), and *ampC *(the gene encoding β-lactamase) genes. These proteins have a D,Dcarboxypeptidase activity and are involved in the formation of precursors of the peptidoglycan membrane layer affecting the degree of cross-linking of cell wall peptidoglycans by regulating the number of components available for crosslinking [66]. Reductive division is a form of cell adaptation to adverse environmental conditions. Reductive division allows cells to gain advantages under conditions of starvation due to increased the surface to volume ratio, producing more spherical cells. It is worth noting that reductive division is not induced by starvation and is determined by starvation onset at the time of active cell division.

The formation of dwarf cells, unlike reductive division, is activated by starvation. This process is characterized by a steady decrease in the size of the cells due to degradation not only of endogenous resources, but also of the cell wall, particularly the cytoplasmic membrane and the cell wall. In some Gram-negative bacteria such as *E. coli*, in the stationary phase the outer membrane is not degraded and is not compressed, like the inner one, which leads to an increase in the periplasmic space [67].

A distinctive feature of the adaptation to the stationary phase is the formation of a cell wall that can efficiently withstand harsh environmental settings. The formation of this intensified barrier includes extensive changes at all levels of bacterial membranes: the inner and outer membranes, periplasm, and peptidoglycans. In the outer membrane, polysaccharide concentration increases, the amount of proteins decreases, and the number of molecular crosslinks between lipoproteins of the outer membrane and the peptidoglycan layer increases. Oligosaccharides, such as trehalose, accumulate in the periplasm to act as osmoprotectants. The peptidoglycan layer (peptidoglycan is a strong and elastic polymer that serves as the stress-bearing component of the bacterial cell wall) increases its thickness. Recently, it has been shown that in the stationary phase, D-amino acids modify the peptidoglycan layer by means of their incorporation into the peptidoglycan polymer. Some significant changes occur in the structure of the inner membrane. The amount of monounsaturated fatty acids falls with simultaneous increase in the proportion of polyunsaturated fatty acids. Unsaturated fatty acids are also converted into cyclopropyl derivatives, and the ratio of the number phosphoglycerine to phosphoethanolamine increases when the cell enters the stationary growth phase. The consequence of all these changes is the formation of a rigid structure of the inner membrane and its reduced fluidity [64].

**Microevolution of bacteria in the stationary phase**

The bacterial population can adapt to adverse conditions in unexpected ways. During the observation of cell survival in the stationary phase, it was discovered that during quite a long incubation of the culture without change of the nutrient medium the growth curve had a characteristic successive rise and fall in the number of viable cells. This phenomenon was defined as the growth advantage in the stationary phase (GASP) phenotype. This behavior of the culture is accounted for by the emergence of mutant cells among the population, which are better adapted to these conditions than the parent strain [1].

GASP-phenotype is mediated by several key mutations which benefit in the stationary phase. One of these mutations leads to reduced activity of σ^38^ , and* rpoS *deletion mutant strains showed no characteristic phenotype. It is supposed that the advantages of this mutation are a result of its pleiotropic effect. This effect may be determined by imbalance in the competition among sigma factors for the RNA polymerase [3].

Another mutation, more exactly a group of mutations that leads to GASP-phenotype, is mutation in the* lrp *and *sgaC *genes, as well as the genomic rearrangement inactivating the *cstA *gene and activating operon* ybeJ-gltJKL*. These mutants actively feed on the amino acids debris entering the environment from the dead cells. A genomic rearrangement leads to the deactivation of a gene encoding oligopeptide permease and activation of operon encoding annotated transporterproteins of glutamic and aspartic acids. Thus, due to lost ability to degrade oligopeptides the cell acquires enhanced ability to feed on monomeric amino acids entering the medium from dead cells. After additional incubation in cells with a mutation in the *rpoS *gene, the* bgl *operon is activated, which leads to the appearance of a population that can use aryl-β-glycosides arbutin and salicin as a resource [3].

Interestingly, in the stationary phase a population of cells appears with a viable but nonculturable (VBNC) phenotype. This phenotype manifests as a response to a variety of stresses and occurs in many bacteria. The molecular nature of the mechanism of VBNC-phenotype has not been identified, but it is clear that it goes beyond a single regulatory pathway and includes a global change in cell metabolism. A feature of the VBNC-phenotype is the huge reduction in metabolism and changes in cell morphology. Probably, in this way the cell is trying to cope with stress via “hibernation” and fence itself from the harsh environment by an impermeable barrier. The mechanism of exit from this state is little known [68].

**Antibiotic resistance in the stationary phase**

Rapid growth in the frequency of use of antibiotics against bacterial infections results in rapid emergence of bacterial strains resistant to antibiotics. Therefore, an “arms race” with bacteria has become one of the most important issues in modern medicine. A search for newer and newer antibiotics is necessary in order to overcome the problem of resistance for a while. One of the directions in this “arms race” involves the study of the cellular mechanisms that confer resistance, instead of a search for new antibiotics, since through suppression of these mechanisms it is possible to overcome the problem of bacterial resistance to antibiotics.

It has long been observed that bacterial resistance to the action of different classes of antibiotics increases significantly under starvation. As already mentioned, the cell cycle and all stages of the genetic information implementation are suppressed in the stationary phase. Accordingly, antibiotic resistance has been mostly accounted for by the absence of growth of bacteria during starvation [69].

The molecular mechanism of bacterial resistance to antibiotics of different classes in the stationary growth phase during growth arrest has relatively recently been identified. It has been shown that upon deactivation of the *relA *and *spoT *genes (which makes it impossible to develop a stringent response), bacterial resistance to antibiotics decreases significantly and the intracellular concentration of hydroxyl radicals increases. Since it is known that the lethal action of almost all classes of antimicrobials is eventually determined by the accumulation of reactive oxygen species in the cell [70], the authors of the research decided to assess the level of catalase activity in the cells, which proved to be significantly reduced. Thus, it was shown that active cell response to stress, rather than the absence of growth, is important in tolerance to antibiotics in the stationary phase [71].

## CONCLUSIONS

The cell deals with survival in harsh settings in various ways. For protection against mechanical damage and stress factors, the cell wall is strengthened and rebuilt and the shape of cells changes. In turn, the nucleoid becomes condensed and is included in the nucleoprotein complex to protect it from damage.

To save resources, the translation process is inhibited in particular by downregulation of the expression of genes encoding components of the protein biosynthetic machinery. Particularly interesting is the variety of regulatory pathways through which translation is suppressed. It is the translational apparatus, as the most energy-consuming process, that is the key member of stress signal transmission to other components of the cell. Depending on the extent of starvation, the cell passes the pathway from reduced expression of ribosomal operons to complete suppression of translation and degradation of ribosomes.

The cell’s ability to use an alternative sigma factor for the regulation of gene expression of stress response is also important. A complex system of regulation of synthesis and stability of the sigma factor allows the cell to respond immediately to the occurrence of stress and quickly return to normal growth.

It becomes clear that the transition to the stationary growth phase is a natural defense mechanism of bacterial culture to cope with stress and starvation. Under these conditions, the cell structure changes at all levels of the organization directed at the survival of both individual cells and the whole population.

## Abbreviations

GASP: the growth advantage in stationary phase (GASP) phenotype
UTR: untranslated region
TLD: tRNA-like domain
MLD: mRNA-like domain
tmRNA: transport and messenger RNA
PCD: programmed cell death
TA: toxin-antitoxin
QS: bacterial intercellular communication system
VBNC: viable but nonculturable bacteria

## References

[1] Finkel S.E. (2006). Nat. Rev. Microbiol..

[2] Pin C., Baranyi J. (2008). Appl. Environ. Microbiol..

[3] Llorens J.M.N., Tormo A., Martínez-García E. (2010). FEMS Microbiology Rev..

[4] Luijsterburg M.S., Noom M.C., Wuite G.J.L., Dame R.T. (2006). J. Structural Biol..

[5] Azam T.A., Iwata A., Nishimura A., Ueda S., Ishihama A. (1999). Journal of Bacteriology.

[6] Nair S., Finkel S.E. (2004). Journal of Bacteriology.

[7] Almirón M., Link A.J., Furlong D., Kolter R. (1992). Genes Dev..

[8] Wolf S.G., Frenkiel D., Arad T., Finkel S.E., Kolter R., Minsky A. (1999). Nature.

[9] Ilari A., Ceci P., Ferrari D., Rossi G.L., Chiancone E. (2002). J. Biol. Chem..

[10] Cosgriff S., Chintakayala K., Chim Y.T.A., Chen X., Allen S., Lovering A.L., Grainger D.C. (2010). Mol. Microbiol..

[11] Kahramanoglou C., Prieto A.I., Khedkar S., Haase B., Gupta A., Benes V., Fraser G.M., Luscombe N.M., Seshasayee A.S.N. (2012). Nat. Commun..

[12] Ishihama A. (2000). Annu. Rev. Microbiol..

[13] Reznikoff W.S., Siegele D.A., Cowing D.W., Gross C.A. (1985). Annu. Rev. Genet..

[14] Maciąg A., Peano C., Pietrelli A., Egli T., Bellis G.D., Landini P. (2011). Nucleic Acids Research.

[15] Arnosti D.N., Chamberlin M.J. (1989). Proc. Natl. Acad. Sci. USA..

[16] Hunt T.P., Magasanik B. (1985). Proc. Natl. Acad. Sci. USA..

[17] Straus D.B., Walter W.A., Gross C.A. (1987). Nature.

[18] Rhodius V.A., Suh W.C., Nonaka G., West J., Gross C.A. (2005). PLoS Biol..

[19] Enz S., Braun V., Crosa J.H. (1995). Gene..

[20] Weber H., Polen T., Heuveling J., Wendisch V.F., Hengge R. (2005). Journal of Bacteriology.

[21] Gaal T., Ross W., Estrem S.T., Nguyen L.H., Burgess R.R., Gourse R.L. (2001). Mol. Microbiol..

[22] Typas A., Hengge R. (2006). Mol. Microbiol..

[23] Mika F., Hengge R. (2005). Genes Dev..

[24] Lange R., Hengge-Aronis R. (1994). Genes Dev..

[25] Mukhopadhyay S., Audia J.P., Roy R.N., Schellhorn H.E. (2000). Mol. Microbiol..

[26] Gentry D.R., Hernandez V.J., Nguyen L.H., Jensen D.B., Cashel M. (1993). Journal of Bacteriology.

[27] Cunning C., Brown L., Elliott T. (1998). Journal of Bacteriology.

[28] Brescia C.C., Kaw M.K., Sledjeski D.D. (2004). J. Mol. Biol..

[29] Lease R.A., Belfort M. (2000). Proc. Natl. Acad. Sci. USA..

[30] Muffler A., Fischer D., Hengge-Aronis R. (1996). Genes Dev..

[31] Zhang A., Altuvia S., Tiwari A., Argaman L., Hengge–Aronis R., Storz G. (1998). EMBO J..

[32] Majdalani N., Hernandez D., Gottesman S. (2002). Mol. Microbiol..

[33] Trotochaud A.E., Wassarman K.M. (2004). Journal of Bacteriology.

[34] Jishage M., Kvint K., Shingler V., Nyström T. (2002). Genes Dev..

[35] Jishage M., Ishihama A. (1999). Journal of Bacteriology.

[36] Gyaneshwar P., Paliy O., McAuliffe J., Jones A., Jordan M.I., Kustu S. (2005). Proc. Natl. Acad. Sci. USA..

[37] Typas A., Barembruch C., Possling A., Hengge R. (2007). EMBO J..

[38] Schweder T., Lee K.H., Lomovskaya O., Matin A. (1996). Journal of Bacteriology.

[39] Santos J.M., Freire P., Mesquita F.S., Mika F., Hengge R., Arraiano C.M. (2006). Mol. Microbiol..

[40] Bougdour A., Wickner S., Gottesman S. (2006). Genes Dev..

[41] Zhou Y., Gottesman S. (2006). Journal of Bacteriology.

[42] Zgurskaya H.I., Keyhan M., Matin A. (1997). Mol. Microbiol..

[43] Tani T.H., Khodursky A., Blumenthal R.M., Brown P.O., Matthews R.G. (2002). Proc. Natl. Acad. Sci. USA..

[44] Zinser E.R., Kolter R. (2000). Journal of Bacteriology.

[45] Calvo J.M., Matthews R.G. (1994). Microbiol. Rev..

[46] Gottesman S. (2005). Trends Genet..

[47] Majdalani N., Cunning C., Sledjeski D., Elliott T., Gottesman S. (1998). Proc. Natl. Acad. Sci. USA..

[48] Johansen J., Rasmussen A.A., Overgaard M., Valentin-Hansen P. (2006). J. Mol. Biol..

[49] Borek E., Rockenbach J., Ryan A. (1956). Journal of Bacteriology.

[50] Cashel M., Kalbacher B. (1970). J. Biol. Chem..

[51] Boutte C.C., Crosson S. (2013). Trends Microbiol..

[52] English B.P., Hauryliuk V., Sanamrad A., Tankov S., Dekker N.H., Elf J. (2011). Proc. Natl. Acad. Sci. USA..

[53] Murray D.K., Bremer H. (1996). J. Mol. Biol..

[54] Barker M.M., Gaal T., Josaitis C.A., Gourse R.L. (2001). J. Mol. Biol..

[55] Starosta A.L., Lassak J., Jung K., Wilson D.N. (2014). FEMS Microbiol. Rev..

[56] Zvereva M.I., Ivanov P.V., Teraoka Y., Topilina N.I., Dontsova O.A., Bogdanov A.A., Kalkum M., Nierhaus K.H., Shpanchenko O.V. (2001). J. Biol. Chem..

[57] Neubauer C., Gillet R., Kelley A.C., Ramakrishnan V. (2012). Science..

[58] Chadani Y., Ono K., Ozawa S., Takahashi Y., Takai K., Nanamiya H., Tozawa Y., Kutsukake K., Abo T. (2010). Mol. Microbiol..

[59] Chadani Y., Ono K., Kutsukake K., Abo T. (2011). Mol. Microbiol..

[60] Polikanov Y.S., Blaha G.M., Steitz T.A. (2012). Science..

[61] Zhang J., Zhang Y., Inouye M. (2003). J. Biol. Chem..

[62] Amitai S., Kolodkin-Gal I., Hananya-Meltabashi M., Sacher A., Engelberg-Kulka H. (2009). PLoS Genet..

[63] Moll I., Engelberg-Kulka H. (2012). Trends Biochem. Sci..

[64] Nyström T. (2004). Annu. Rev. Microbiol..

[65] Akerlund T., Nordström K., Bernander R. (1995). Journal of Bacteriology.

[66] Santos J.M., Lobo M., Matos A.P.A., De Pedro M.A., Arraiano C.M. (2002). Mol. Microbiol..

[67] Reeve C.A., Bockman A.T., Matin A. (1984). Journal of Bacteriology.

[68] Hayes C.S., Low D.A. (2009). Curr. Opin. Microbiol..

[69] Levin B.R., Rozen D.E. (2006). Nat. Rev. Microbiol..

[70] Kohanski M.A., Dwyer D.J., Hayete B., Lawrence C.A., Collins J.J. (2007). Cell..

[71] Nguyen D., Joshi-Datar A., Lepine F., Bauerle E., Olakanmi O., Beer K., McKay G., Siehnel R., Schafhauser J., Wang Y. (2011). Science..

